# Metalloid–Organic Intermolecular Complexes with Charge State-Controlled Conformations

**DOI:** 10.3390/molecules29071635

**Published:** 2024-04-05

**Authors:** Fedor Y. Naumkin

**Affiliations:** Faculty of Science, Ontario Tech University/UOIT, Oshawa, ON L1G 0C5, Canada; fedor.naumkin@uoit.ca

**Keywords:** intermolecular complexes, metal–organic compounds, ions, isomerization, ab initio calculations

## Abstract

Shape alterations of molecular systems, induced by their (electric) charging/discharging, could facilitate useful electronic and/or mechanical functions in molecular-scale devices and machines. The present study reports structures, stabilities, charge distributions, and IR spectra for a group of complexes of a main-group metalloid (boron) atom with hydrocarbon molecules. The considered systems include the smallest species demonstrating the basic principle of operation, as well as their size-extended analogues, generalizing it to larger counterparts based on such units. The system geometries vary considerably between neutral and ionic counterparts and exhibit two–three typical conformations related to twisting by up to about 90 degrees. The predicted structures correlate with specific infrared spectra, which can enable their experimental identification and transformation tracking. The above-mentioned characteristics suggest the potential utility of such systems for intermolecular switches, with the possible spectral monitoring of their functioning.

## 1. Introduction

Metal–organic complexes are characteristic representatives of and are at the very heart of inorganic chemistry. They constitute an enormous variety of systems with numerous applications, such as novel materials with unique optical and magnetic properties, catalyses, and ways of utilizing energy (e.g., sun radiation), etc. Transition metals dominate in this area, while the present work contributes to the increasing volume of studies involving main-group metals.

Specifically, here, the focus is on boron complexes with the smallest and larger hydrocarbon molecules. Boron chemistry is a broad and versatile field, in particular due to B standing next to C in the periodic table; thus, organoboron species have properties that match and enrich those of carbon-based species. In particular, boron-carbon based compounds form a large family of systems, with these atoms being bonded to each other. Further, multiple chemical reactions deal with the formation, breaking, and/or transformation of such bonds. In particular, relevant reviews [1,2] (see also the references therein) describe the role of boron in organic synthesis, along with an explosive growth of its applications in materials and pharmaceutical science [3,4]. For instance, some protease inhibitors containing boron are approved medications for dealing with viral infections such as HIV/AIDS, hepatitis C, and COVID-19.

One relatively recent example includes the minimal compounds of B and ethylene (C_2_H_4_) and their shape alterations depending on the system charge state, i.e., upon ionization and electron attachment [5]. Namely, for the B-(C_2_H_4_)_2_ ternary complex, the molecules attached on opposite sides of the atom would significantly change their relative orientation within the range from parallel (for cation) to intermediate (for neutral species) to perpendicular (for anion), with the pairs of B-C bonds twisting accordingly. So, the system behaves like a molecular structural switch controlled by electric charge, with hypothetical practical uses in molecular electronics and machinery, both being areas experiencing a hyperactive rise [6,7,8,9] (see the references therein as well).

The aim of the present work was to expand the above study and check the properties and behaviour of analogous systems with acetylene components (having triply bonded dicarbon, unlike in ethylene) as well as those of extended systems with larger molecules, such as those with H atoms replaced by CH_3_ groups while keeping the same central structural unit (CC-B-CC). Specifically, here, the butyne (C_4_H_6_) and butene (C_4_H_8_) species are considered. The related goal was to test the generality of the charge-induced geometry variations for boron–hydrocarbon complexes with similar bond patterns. The present work confirms such common features of the small and larger systems and compares them in terms of stability, charge distributions, and IR spectra. The latter properties can also serve as a means for the experimental verification of the system formation and transformations.

This study could thus be considered as related to merging the fields of boron chemistry and molecular devices. An earlier directly relevant review [10] addressed, both experimentally and theoretically, reactions of B with neutral hydrocarbons (including methane, ethylene, acetylene, and others), leading to the formation of organoboron compounds. The present study extends the scope of the subject to other molecules, ternary systems, and ionic derivatives.

## 2. Results and Discussion

First, the smallest relevant systems are considered, with hydrogen-terminated (double- and triple-bonded) C=C and C≡C units. Then, their methylated structural extensions with longer carbon skeletons are included. For each group, neutral and ionic species are investigated in terms of a set of parameters, as described below.

### 2.1. Minimal Systems

The smallest considered systems include binary and ternary complexes of B atoms with one or two acetylene and ethylene molecules.

#### 2.1.1. Structures and Stabilities

**Figure 1:**
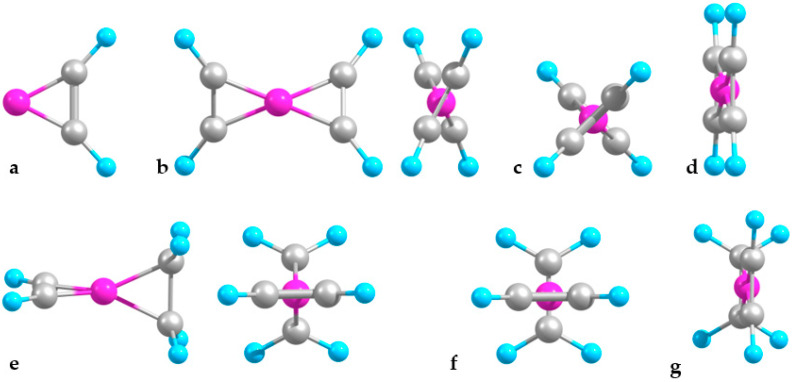
Optimized geometries: (**a**) B-C_2_H_2_, (**b**) B-(C_2_H_2_)_2_ (front and side views), (**c**) B-(C_2_H_2_)_2_^−^, and (**d**) B-(C_2_H_2_)_2_^+^ (side views); (**e**) C_2_H_2_-B-C_2_H_4_ (front side views), (**f**) C_2_H_2_-B-C_2_H_4_^−^, and (**g**) C_2_H_2_-B-C_2_H_4_^+^ (side views).

A boron atom attaches to an acetylene molecule sideways (Figure 1), forming two B-C bonds at the expense of a (broken) C-C π-bond and apparently altering the carbon–atom hybridization from sp to (approximately) sp^2^, hence strongly bending C_2_H_2_ away from B. This binary complex is considerably stable by a few electronvolts (Table 1). The attachment of a second C_2_H_2_ unit symmetrically on the opposite side of B proceeds similarly and results in a structure with the CC units being considerably twisted relative to one another (see Table 1). As a preliminary interpretation, the latter feature could lower the mutual repulsion of the negatively charged CC units. The ternary B-(C_2_H_2_)_2_ complex shows a notable anti-cooperativity in stability, with the second added molecule increasing the binding by only a third (about 1.1 eV) relative to B-C_2_H_2_. Accordingly, the B-C bonds stretch, the C-C bonds shrink, and the molecules partially unbend (Table 1). Such nonadditivity is consistent with the binding ability of (monovalent) B, now shared between two attached molecules, and with their mutual electrostatic repulsion.

The above system geometries resemble those of the previously studied ethylene-based counterparts B-C_2_H_4_ and B-(C_2_H_4_)_2_ [5]. The former is significantly less bound (by about 1 eV) relative to B-C_2_H_2_, while the latter is bound equally compared to B-(C_2_H_2_)_2_ (Table 1). Accordingly, the B-C distances are longer, and the interaction nonadditivity is lower than for the acetylene-based species. The D_e_ values for the binary species slightly exceed those obtained earlier, 3.06 eV for B-C_2_H_2_ [11] and 2.02 eV for B-C_2_H_4_ [12].

Unlike the above complexes with two identical molecules, the C_2_H_2_-B-C_2_H_4_ one is predicted to be maximally twisted (Figure 1), so the attached molecules are perpendicular to each other. This is likely due to weaker C-C interactions between the CC units of different lengths. Still, the hetero-molecular complex is slightly more bound than either homo-molecular counterpart (Table 1). This stronger stabilization appears to be due to the increased B-C_2_H_2_ interaction, as follows from the B-C bonds being shorter, C-C bond longer, and CCH bending larger than in B-(C_2_H_2_)_2_, with opposite relations for the B-C_2_H_4_ fragment relative to that in B-(C_2_H_4_)_2_. Indeed, attaching C_2_H_2_ to B-C_2_H_4_ adds about 2.3 eV of binding as compared to about 1.4 eV for attaching C_2_H_4_ to B-C_2_H_2_.

The corresponding ions of B-(C_2_H_2_)_2_ have analogous overall structures, with B sandwiched in between two molecules which, however, alter their mutual orientation. In the anionic and cationic complexes, the molecules are, respectively, perpendicular and parallel to one another (Figure 1). This could be qualitatively rationalized in terms of the molecules charged more and less negatively, respectively; accordingly, their mutual repulsion is stronger and weaker, leading to their increased and decreased relative twisting.

The stability increases from neutral B-(C_2_H_2_)_2_ to its anion to its cation. In particular, this is a general trend for all the above ternary complexes (Table 1). Curiously, for B-C_2_H_2_, the anion behaves similarly, while the cation is less stable than both the anion and neutral counterparts. As a result, the cationic complexes even show a cooperative nonadditivity of binding. The B-C distance increases for the anion and decreases for the cation in both binary and ternary complexes. The C-C distance in the CC units shows opposite variations for B-C_2_H_2_ but, interestingly, similar variations for B-(C_2_H_2_)_2_.

The ions of B-(C_2_H_4_)_2_ [5] repeat the shape changes exhibited by B-(C_2_H_2_)_2_. Here, another correlation could be suggested to interpret the shape of the anion, namely B-(C_2_H_4_)_2_^−^ being isoelectronic to spiropentane (with central C instead of B) which is known to have perpendicular C_2_H_4_ units [13]. Curiously, an analogous consideration of anionic B-(C_2_H_2_)_2_ seems to fail, since isoelectronic spiropentadiene differs in shape (with parallel C_2_H_2_ units). The stabilization relative to neutral B-(C_2_H_4_)_2_ is stronger for the anion and weaker for the cation compared to the B-(C_2_H_2_)_2_ case, so the two ternary ions are nearly equally stable (Table 1). Additionally, now it is the ternary anion which is bound cooperatively. The B-C bonds shrink for the anion and stretch for the cation, opposite to the B-(C_2_H_2_)_2_ case. The C-C bonds change in counterphase and are thus similar to those in B-(C_2_H_2_)_2_. The B-C_2_H_4_ complex is also stabilized by anionization, while ionization reshapes it via the insertion of B inside the stretched CC unit (making a linear CBC core). The latter system is therefore not considered further here. For B-C_2_H_4_, anionization increases the B-C bond length and decreases the C-C bond length, similar to the variations in B-C_2_H_2_ but oppositely to those in B-(C_2_H_4_)_2_.

The C_2_H_2_-B-C_2_H_4_ hybrid, upon ionization, transforms similar to the homo-molecular counterparts, with the molecules becoming parallel to one another, while anionization leaves the shape of the complex (with mutually perpendicular molecules) unchanged (Figure 1). The system stability rises from neutral to anion to cation, thus reproducing the variations in B-(C_2_H_2_)_2_, even though the increments are somewhat smaller. The related B-C and C-C distance alterations in the C_2_H_2_ and C_2_H_4_ components follow those in B-(C_2_H_2_)_2_ and B-(C_2_H_4_)_2_, respectively, except for the C-C distance in C_2_H_2_, decreasing from the neutral species to the anion.

For the binary species, the BSSE correction reduces the dissociation energies by about 0.5 ± 0.1 eV, being maximal for the anions. The values increase to about 0.6–0.7 eV for the ternary systems.

#### 2.1.2. Charge Distributions

The B atom is predicted to be positively charged in all neutral systems studied (Table 2) within the range of 0.45–0.70 e. The charge on B is smallest for the acetylene-based and largest for the ethylene-based species and slightly reduces from the binary to ternary complexes. The latter feature can be related directly to the competition between the two attached molecules (pulling the charge in opposite directions) and inversely to the B-C separations (longer in the ternary systems) determining the charge transfer distance.

In the respective anions and cations, the B atom becomes, respectively, less and more positive, up to near-zero or even negative charges in the binary anionic complexes and up to a full unit charge in cationic B-C_2_H_2_. Curiously, B-(C_2_H_2_)_2_ shows a counter-intuitive exception here, with B being less positive for the cation than the anion.

#### 2.1.3. Ionization Energies and Electron Affinities

The energy differences between the cation and neutral system and between the latter and anion conventionally define the ionization energy (IE) and electron affinity (EA), respectively. Their calculated values for the present systems are presented in Table 3. Such values for the B atom are reported as well and can be seen to match well with the experimental data, offering further validation of the employed theoretical approach.

The IE values are lower than IE(B) for all systems except B-C_2_H_2_. This could be readily related to the D_e_ values being larger for the cations than for the corresponding neutrals, while this relation is opposite only for B-C_2_H_2_ (Table 1). The IEs are lower for the ternary systems compared to the binary systems. Opposite to the situation for the binary species, the acetylene-based ternary system has lower IE compared to the ethylene-based one, while the value for the hetero-molecular case is intermediate between the two homo-molecular counterparts.

In turn, the EA values for all systems are considerably higher than EA(B), consistent with the higher stabilities of the anions relative to the neutrals. The ternary systems are about triply more electrophilic than the binary ones. The ethylene-based species have higher EAs compared to the acetylene-based ones, while the hetero-molecular system has a lower EA compared to either homo-molecular analogue.

#### 2.1.4. Simulated IR Spectra

**Figure 2:**
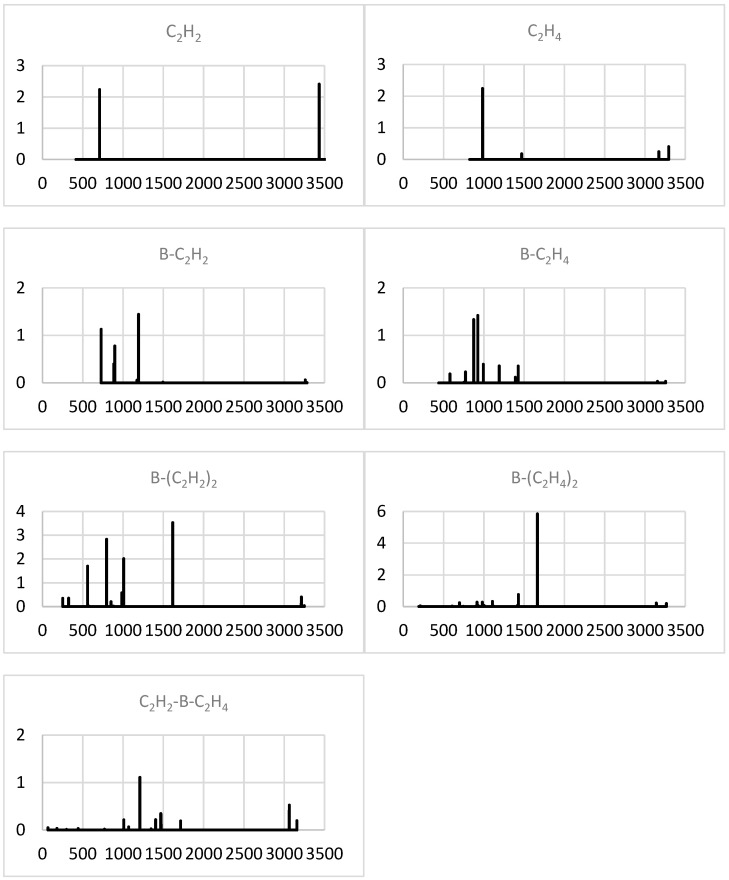
Simulated IR spectra (intensity in (D/Å)^2^ vs. frequency in cm^−1^) of the minimal systems studied.

Upon the formation of a binary complex with B, the intense high-frequency (C-H stretch related) IR band of C_2_H_2_ is apparently cancelled (Figure 2), while its low-frequency (bending) doublet is expanded by a group of blue-shifted (by up to 500 cm^−1^) lines. The most intense one of the latter (near 1200 cm^−1^) corresponds to a B-C_2_H_2_ axial stretch. For C_2_H_4_, the dominating low-frequency (C-C-H bending) band splits into anti- and symmetric modes and red-shifts upon such a complexation.

The addition of a second acetylene or second ethylene unit leads to the appearance of a dominant mid-range (near 1600 cm^−1^) band associated with an antisymmetric C_2_H_2/4_-B-C_2_H_2/4_ axial stretch, while the lower-frequency part of the spectrum is intensified and red-shifted in the former but suppressed in the latter case. The hetero-molecular ternary complex in effect mainly preserves the most intense band of B-C_2_H_2_ (near 1200 cm^−1^) and even somewhat recovers its (red-shifted) C-H stretch component.

**Figure 3:**
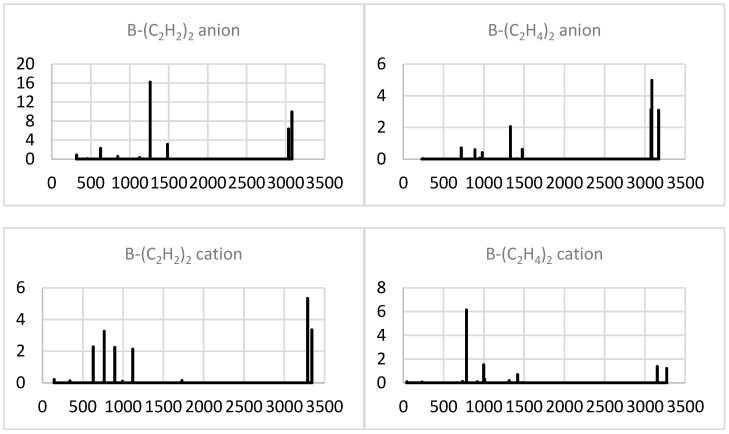
Simulated IR spectra (intensity in (D/Å)^2^ vs. frequency in cm^−1^) of the minimal system ions.

The anionization of B-(C_2_H_2_)_2_ produces a very intense band near 1300 cm^−1^ due to (red-shifted) antisymmetric axial stretching and strong high-frequency (antisymmetric C-H stretching) bands (Figure 3). The latter also dominate for cationic B-(C_2_H_2_)_2_, which preserves the low-frequency group of lines but not the band dominating in the neutral counterpart. The predominant band of B-(C_2_H_4_)_2_ is also removed in both ions, and their spectra exhibit intense high- and low-frequency bands for the anion and cation, respectively. For the cation, the most intense band, near 800 cm^−1^, corresponds to the (strongly red-shifted) antisymmetric axial stretch.

### 2.2. Extended Systems

The following systems are structural derivatives of the above minimal species, with two hydrogen atoms replaced by methyl radicals.

#### 2.2.1. Structures and Stabilities

**Figure 4:**
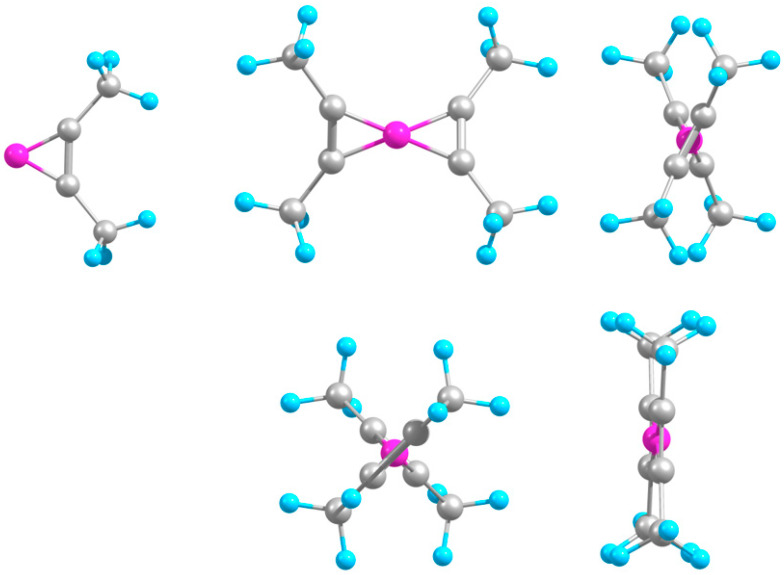
Optimized geometries. Top row (left to right): B-C_4_H_6_, B-(C_4_H_6_)_2_ (front and side views); second row: B-(C_4_H_6_)_2_^−^ and B-(C_4_H_6_)_2_^+^ (side views).

Both B-C_4_H_6_ and B-(C_4_H_6_)_2_ systems geometrically resemble the acetylene-based counterparts (Figure 4), including a considerable twist (by about same angle) between the middle CC units in the ternary system, as expected due to the identical B-molecule bonding scheme. Accordingly, the binding energies and the B-C and (middle hereafter) C-C bond lengths are almost unchanged relative to the smaller counterparts (Table 4). In particular, this is also consistent with a similar invariability of stability from boron–acetylene to boron–methylacetylene when a single H is replaced by CH_3_, as predicted earlier [14]. The dimethylacetylene (coinciding with the presently studied C_4_H_6_) has also been previously studied in terms of making compounds with B [15], including its structure (shown in Figure 4), though with no stability characterization.

The corresponding ions are also structurally similar to the smaller analogues, with the molecules being perpendicular and parallel in the anionic and cationic ternary complexes, respectively. The binding energies increase from neutral to anionic to cationic complexes for both one and two attached molecules, stronger for the latter case and leading to almost additive stabilization for the ternary cation. This generally repeats the case of neutral counterparts except for the opposite relation between the relative stabilities of the binary ionic complexes. The B-C and C-C bonds are slightly longer in the ternary compared to the respective binary systems and vary with the charge state similarly for both cases.

**Figure 5:**
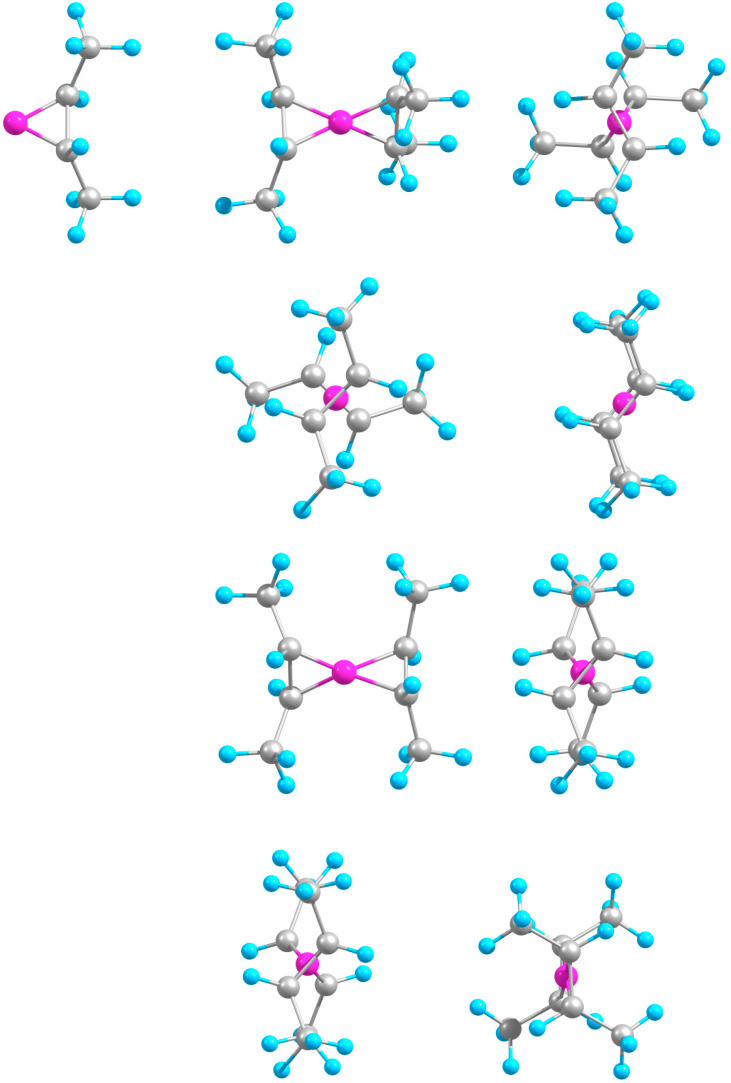
Optimized geometries. Top row (left to right): B-C_4_H_8_, cis B-(C_4_H_8_)_2_ (front and side views); second row: cis B-(C_4_H_6_)_2_^−^ and B-(C_4_H_6_)_2_^+^ (side views); third row: trans B-(C_4_H_8_)_2_ (front and side views); bottom row: trans B-(C_4_H_8_)_2_^−^ and B-(C_4_H_8_)_2_^+^ (side views).

Similarly, B-C_4_H_8_ and B-(C_4_H_8_)_2_ also have geometries analogous to those of the ethylene-based species. Here, the ternary system exhibits two different conformers, with two attached molecules oriented in phase or antiphase (Figure 5), whose cases are labelled cis and trans, respectively. The middle CC units are twisted at an angle which is close to that in B-(C_4_H_6_)_2_ for the cis but larger for the trans conformer (Table 4). Visually, the overall CCCC skeletons look more aligned for the trans case. The cis and trans conformers are nearly equally stable to dissociation, while both these and the binary complex are slightly less stable (by about 0.3–0.4 eV) than the respective ethylene-based counterparts. While B-C_4_H_8_ is considerably less stable (by about 1.3 eV) than B-C_4_H_6_, the difference reduces to about 0.4 eV for the corresponding ternary species, similar to the smaller systems. The B-C and C-C bonds are again slightly stretched and shrunk, respectively, in the ternary relative to binary systems. The trans case, however, shows some asymmetry, with one molecule being slightly farther away from B and, accordingly, shorter than the other.

The respective ions of the cis conformer reproduce the relative orientations of the middle CC units of the molecules found for the smaller (ethylene-based) counterparts—perpendicular for the anion and parallel for the cation—while for the trans case, the angles are slightly different (Table 4). Accordingly, the overall orientations of the CCCC skeletons of the ions are also perpendicular and parallel for the cis but opposite for the trans cases (Figure 5). Unlike for the smaller (ethylene-based) counterparts, the trans anion remains almost unchanged from the corresponding neutral species. Similar to the C_4_H_6_-based species, the binding energies increase from the neutral system to anion to cation more strongly for the ternary complexes (and equally for the cis and trans cases). The ternary anion exhibits a cooperative binding, similar to the ethylene-based analogue. While the cation of B-C_4_H_8_ features B inserted into the middle CC unit, similar to the corresponding smaller (ethylene-based) system, being therefore also dropped from further consideration. In anionic B-(C_4_H_8_)_2_, the attached molecules somewhat converge towards B and stretch, oppositely to the variations in anionic B-C_4_H_8_, and then evolve reversely in the ternary cation. This is similar to the binary C_4_H_6_-based complexes but different from the ternary ones. These alterations resemble those for the smaller analogues.

**Figure 6:**
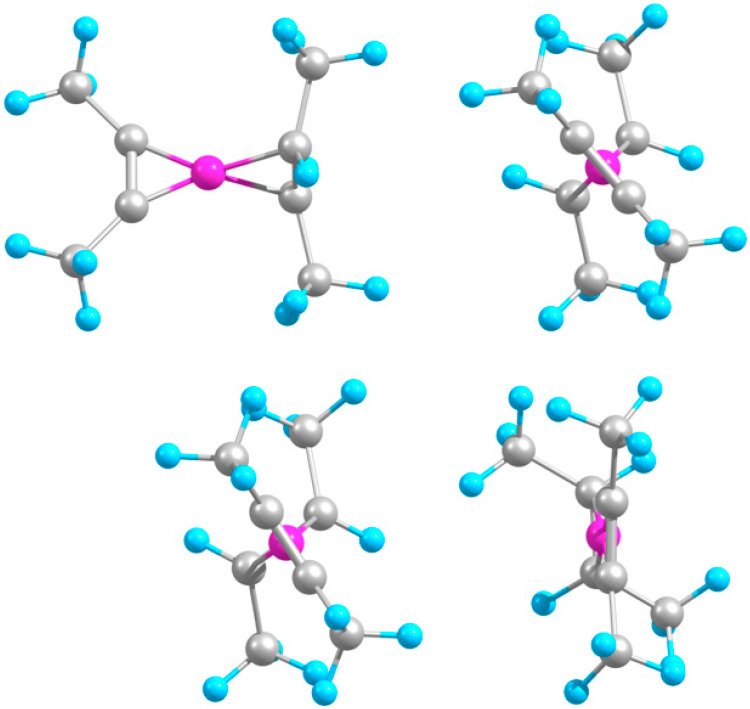
Optimized geometries. **Top**: C_4_H_6_-B-C_4_H_8_ (front and side views); **Bottom**: C_4_H_6_-B-C_4_H_8_^−^ and C_4_H_6_-B-C_4_H_8_^+^ (side views).

In the hetero-molecular C_4_H_6_-B-C_4_H_8_ system, the middle CC units are near-perpendicular (Figure 6), as in the smaller counterpart (with acetylene and ethylene). The complex is more stable than its homo-molecular analogues (Table 4), also similar to the situation for the respective smaller species. The C_4_H_6_ unit is closer than C_4_H_8_ to B, and for the former, the B-C and C-C bonds are, respectively, shorter and longer than in B-(C_4_H_6_)_2_, while for the latter, they are intermediate between those in cis and trans B-(C_4_H_8_)_2_. Both B-C and C-C bonds are also marginally longer than in the smaller counterpart (C_2_H_2_-B-C_2_H_4_).

The anionic derivative of C_4_H_6_-B-C_4_H_8_ approximately preserves the near-perpendicular orientation of the middle CC units, while they become near-parallel in the cation (Figure 6). Similar to the homo-molecular analogues, the stabilities increase upon anionization and more so upon ionization (Table 4). For both ions, the binding energies are intermediate between those for B-(C_4_H_6_)_2_ and B-(C_4_H_8_)_2_, unlike for the neutral systems. The C_4_H_6_ unit slightly withdraws from B in the anion and returns even closer in the cation, while C_4_H_8_ behaves in counterphase while remaining farther from B.

The BSSE energies are about 0.4 eV for all binary systems, comparable to those for the minimal counterparts. The values double for the ternary systems to about 0.7–0.8 eV.

#### 2.2.2. Charge Distributions

The extended systems show general similarities in the distributions of charges as compared to the smaller analogues (Table 2). In particular, the B atom is positive in all systems except B-C_4_H_6_ (where it is slightly negative) and B-C_4_H_8_ (where it is essentially neutral). The largest charge on B is found in cationic B-(C_4_H_8_)_2_ (both conformers) and B-C_4_H_6_, although the smaller (ethylene- and acetylene-based) counterparts show inverse relative B charges between the binary and ternary species. The extended systems consistently exhibit higher B charges, with the only exception being the B-C_4_H_6_ vs. B-C_2_H_2_ cations. In all extended ternary complexes containing C_4_H_8_, the charge on B expectedly decreases from neutral to anion and then increases in the cation, while the variation is opposite for the B-(C_4_H_6_)_2_ case, similar to the charge evolutions in the smaller analogues.

#### 2.2.3. Ionization Energies and Electron Affinities

The IE and EA values for the extended systems are lower than for the corresponding smaller counterparts, except for the opposite relation between B-C_4_H_8_ and B-C_2_H_4_ (Table 3). The relations between the values for the extended systems reproduce those for their smaller analogues.

#### 2.2.4. Simulated IR Spectra

The calculated IR intensity spectra for C_4_H_6_ (butyne) and C_4_H_8_ (butene) exhibit a few distinct bands, including the most intense ones near 3000 cm^−1^ and duller ones near 1500 and (for butene) 1000 cm^−1^. The positions and relative intensities of these bands match well with the available experimental data [13].

**Figure 7:**
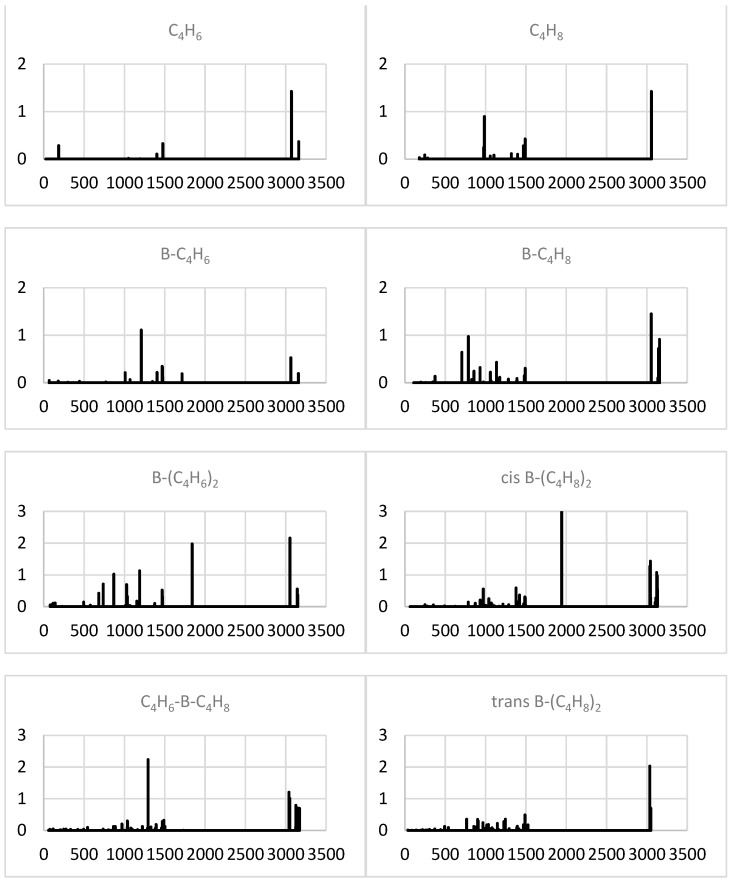
Simulated IR spectra (intensity in (D/Å)^2^ vs. frequency in cm^−1^) of the extended system studied.

The attachment of B weakens the high-frequency (C-H stretching) band for C_4_H_6_ while adding weaker satellites to it for C_4_H_8_ (Figure 7). The new bands appear, including the most intense one near 1200 cm^−1^ for the butyne complex (corresponding to its axial stretch) and the bands between 500 and 1000 cm^−1^ for the butene complex, with the latter effectively red-shifting the original (C-C-H bending) band of the free molecule near 1000 cm^−1^.

In the ternary systems, the high-frequency band recovers its intensity in the di-butyne complex, and new intense bands (stronger and slightly blue-shifted for the cis di-butene complex) appear near 2000 cm^−1^ due to the antisymmetric–stretching axial vibrations. In addition, the di-butyne complex acquires a group of lines between 500 and 1000 cm^−1^, while a similar part of the spectrum diminishes in the cis di-butene complex. The latter is also valid for its trans counterpart, which, however, lacks an intense mid-range band, perhaps due to the asymmetry, which allows one to distinguish the two conformers. The IR spectrum of the hetero-molecular complex is dominated by an intense (asymmetric-stretch) band near 1300 cm^−1^, which differentiates this system from both homo-molecular counterparts, and is contributed by the high-frequency bands. For clarity, the dominating band is associated with the system’s axial oscillations between the following formal structures: C_4_H_6_-B---C_4_H_8_ ↔ C_4_H_6_---B-C_4_H_8_.

**Figure 8:**
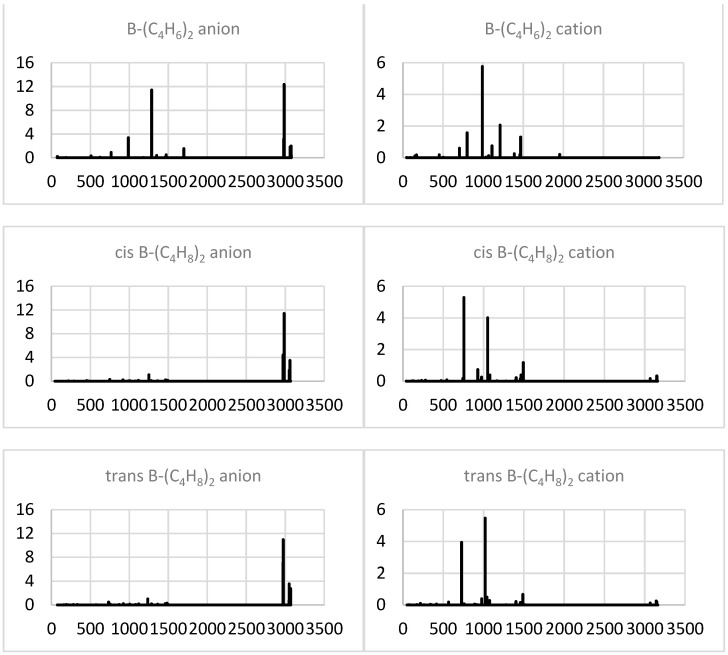
Simulated IR spectra (intensity in (D/Å)^2^ vs. frequency in cm^−1^) of the extended system ions.

The anionization of both homo-molecular ternary complexes considerably focuses their IR spectra, which are dominated by narrow high-frequency (C-H stretching) bands (Figure 8). The di-butyne system also shows a comparably intense band near 1300 cm^−1^, associated with the (red-shifted) antisymmetric–stretch mode. The cations, instead, preserve and intensify the group of bands in the lower frequency range between 500 and 1500 cm^−1^, including the antisymmetric–stretching band near 1000 cm^−1^, the most intense (and still further red-shifted) band for the di-butyne case. The di-butene systems add another intense band near 800 cm^−1^ (due to an antisymmetric stretch as well, but with no accompanying CH_3_ group rocking), which is most intense for the cis conformer. For the trans di-butene conformer, the spectra of the ions are very similar to those for the cis counterpart (especially for the anions), except for the interchanged relative intensities of the two intense (low-frequency) bands for the cations.

## 3. Computational Methods

The present computational study addresses a series of polyatomic intermolecular systems with components interacting both covalently and (especially for extended structures) noncovalently. Accordingly, a consistent MP2 (second-order Moller–Plessett perturbation theory) level approach with aug-cc-pVTZ basis sets [16] for all atoms has been used as a reasonable compromise between the computing time and accuracy (including the reliable handling of relevant dispersion interactions and considerable charge transfer). In particular, spin-unrestricted Hartree–Fock calculations have been used for open-shell systems. Basis set superposition error (BSSE) corrections have been applied via a conventional counterpoise technique [17]. In particular, previous calculations of identical accuracy for (composition-analogous) main-group-metal/benzene complexes [18] showed a very close match to corresponding results produced at a (more accurate) CCSD-T level. In addition, the most relevant (B-C) interactions are obviously involved in the BC diatom, which has been characterized and tested thoroughly against experimental data previously [5]. The above theoretical framework has been employed by means of the NWChem ab initio suite of programs [19].

All system geometries have been fully optimized, and energy minima have been verified via all-real vibrational frequencies. Encountered transition states have been reoptimized using corresponding eigenvectors. For extended systems, optimizations have been carried out with the (reduced) aug-cc-pVDZ basis sets, followed by single-point energy calculations with the above larger basis sets.

Related IR intensity spectra have also been evaluated using NWChem. In particular, test calculations for constituent small molecules have exhibited a good match to available experimental data (with intense bands near 700 and 3400 cm^−1^ for C_2_H_2_ and intense bands near 1000, 1500, and 3200–3300 cm^−1^ for C_2_H_4_). Further tests for extended molecules have also compared well to relevant experiments, as discussed in Section 2.

Charges on atoms have been obtained using the NPA (natural population analysis) approach [20], which has been implemented in the Janpa software (http://janpa.sourceforge.net) [21].

## 4. Conclusions

A series of complexes of a B atom with one and two hydrocarbon molecules were investigated at a consistent theoretical level (MP2/aug-cc-pVTZ + BSSE) in terms of structures, stabilities, charge distributions, and IR spectra. These properties were also predicted for the anionic and cationic derivatives of the systems, with attention being paid to their reshaping upon anionization/ionization. The molecules studied included minimal dicarbon species such as C_2_H_2_ and C_2_H_4_, as well as their tetracarbon extensions (with methyl groups replacing two hydrogens) C_4_H_6_ and C_4_H_8_. Both homo- and hetero-molecular ternary complexes were considered, with two conformers for the C_4_H_8_ case.

The B atom can bind sideways to the CC unit (middle one for the larger species), with the second molecule attaching on the other side (symmetrically in the homo-molecular case). The related re-hybridization of the C atoms bends the molecules. The extended systems are generally similar to the corresponding minimal ones, although with some deviations. In most ternary complexes, the binding is anti-cooperative, with the second molecule being bound less strongly, except for some ions. The significant binding of about 2–4 eV per molecule suggests a ready formation of such systems, at least as direct kinetic products, even if in competition with an isomerization of the binary species, e.g., with H transfer to B [10,22], apparently over significant energy barriers.

In the neutral ternary systems, the molecules are oriented at an intermediate angle (mainly around 60°) to one another, except for in the hetero-molecular species, where the molecules are mutually perpendicular. Electron attachment turns the attached molecules into a perpendicular relative orientation (or keeps it), while ionization makes them parallel, which applies to the middle CC units in the tetracarbon molecules case. The same relative orientations transfer to the entire C_4_H_6_ components, while the orientations can alter (up to inversion) for the C_4_H_8_ ones due the non-flatness of the carbon skeleton in these complexes.

The above transformations are readily reversible for the neutral-anion pair, with the neutral-species geometry recovering upon electron detachment from the corresponding anion. The predicted EA values are moderate (about 1–2 eV), suggesting the charge/discharge to be feasible, and the anions are somewhat more stable to dissociation than the corresponding neutrals, which supports their lifetime. The latter feature is even more favourable for the cations, while the much larger IE values (about 5–6 eV for the extended ternaries) could make such charge/discharge more problematic. Additionally, “returning” an electron to cation normally produces an excited-state species (up to Rydberg one) rather than a ground-state species, and there may well be considerable differences between the geometries of these two states. The relaxation from the excited to the ground state may be complicated, even though one could imagine this to possibly occur, in particular, via a light emission, which would add an interesting, potentially usable (optical effect) behaviour to such a hypothetical switch. Or, perhaps a partial and/or gradual and thus more reversible charge-transfer might be a more realistic option here, maybe via an interaction with a more electronegative entity. At the very least, applications could be limited to neutral-anion switching despite the reduced range of shape alteration.

The simulated IR spectra vary significantly from the free molecules to the binary and ternary complexes (even between conformers) to their ions and can be used for the experimental identification of the species formation and their transformations with the charge state. The variations include the suppression or recovery of the high- (near and above 3000 cm^−1^) and low-frequency bands (around 1000 cm^−1^) characteristic of the free molecules, their spectral shifts and formation of closely located satellites, and the appearance (for homo-molecular ternary systems) or diminishing (in their ions) of new bands in the mid-range (near 2000 cm^−1^). In particular, the IR spectra intensify for ternary homo-molecular complexes and their ions (especially anions).

The above ternary complexes thus appear to be prototype intermolecular systems, by themselves or as units within larger structures or frameworks, capable of offering charge-controlled shape alterations potentially usable for molecular electronics and devices/machines. In addition, their related switching among different geometries (within 90° for relative orientations of the molecular components) could possibly be tracked in terms of rather sensitive IR spectra.

## Figures and Tables

**Table 1:** Equilibrium parameters (dissociation energies, distances, angles) of the minimal systems.

System	Charge State	D_e_/eV	R_e_ (B-C)/Å	R_e_ (C-C)	φ_e_^1^/°	θ_e_ (CC-CC) ^2^
B-C_2_H_2_	neutral	3.41	1.467	1.360	138	
	anion	3.75	1.539	1.336	139	
	cation	3.02	1.391	1.464	132	
B-(C_2_H_2_)_2_	neutral	4.48	1.558	1.309	144	53
	anion	5.89	1.573	1.340	134	90
	cation	7.06	1.538	1.280	156	0
B-C_2_H_4_ *	neutral	2.48	1.519	1.574	138	
	anion	2.95	1.604	1.515	141	
B-(C_2_H_4_)_2_ *	neutral	4.47	1.594	1.475	148 ± 2	60
	anion	6.39	1.574	1.550	133	90
	cation	6.44	1.600	1.438	159	0
C_2_H_2_-B-C_2_H_4_	neutral	4.77	1.492, 1.680	1.347, 1.442	138; 156	90
	anion	5.95	1.568, 1.581	1.331, 1.562	136; 132	90
	cation	6.91	1.487, 1.663	1.310, 1.406	149; 165	0

^1^ CCH bond angle in C_2_H_2_; HCCH axial-folding angle in C_2_H_4_. ^2^ twisting angle between two opposite C-C units. * ref. [5].

**Table 2:** Calculated natural atomic charges in the studied systems.

System	Charge State	q(B)/e	System	Charge State	q(B)/e
B-C_2_H_2_	neutral	0.514	B-C_4_H_6_	neutral	0.543
	anion	−0.221		anion	−0.185
	cation	0.999		cation	0.936
B-(C_2_H_2_)_2_	neutral	0.447	B-(C_4_H_6_)_2_	neutral	0.537
	anion	0.455		anion	0.564
	cation	0.423		cation	0.515
B-C_2_H_4_ *	neutral	0.69	B-C_4_H_8_	neutral	0.762
	anion	−0.04		anion	0.029
B-(C_2_H_4_)_2_ *	neutral	0.59	cis B-(C_4_H_8_)_2_	neutral	0.712
	anion	0.37		anion	0.539
	cation	0.92		cation	1.000
C_2_H_2_-B-C_2_H_4_	neutral	0.541	trans B-(C_4_H_8_)_2_	neutral	0.791
	anion	0.463		anion	0.542
	cation	0.553		cation	1.003
			C_4_H_6_-B-C_4_H_8_	neutral	0.609
				anion	0.549
				cation	0.623

* ref. [5].

**Table 3:** Calculated ionization energies and electron affinities of the studied systems.

System	IE/eV	EA/eV	System	IE/eV	EA/eV
B-C_2_H_2_	8.65	0.58	B-C_4_H_6_	7.60	0.51
B-(C_2_H_2_)_2_	5.76	1.71	B-(C_4_H_6_)_2_	4.62	1.33
B-C_2_H_4_ *	7.37	0.69	B-C_4_H_8_		0.75
B-(C_2_H_4_)_2_ *	6.42	2.21	cis B-(C_4_H_8_)_2_	5.81	2.15
C_2_H_2_-B-C_2_H_4_	6.17	1.46	trans B-(C_4_H_8_)_2_	5.75	2.21
B	8.28 [8.30] ^†^	0.21 [0.28] ^†^	C_4_H_6_-B-C_4_H_8_	5.30	1.26

* ref. [5]. ^†^ experimental values [13].

**Table 4:** Equilibrium parameters (dissociation energies, distances, angles) of the extended systems.

System	Charge State	D_e_/eV	R_e_ (B-C)/Å	R_e_ (C-C) ^1^	φ_e_^2^/°	θ_e_ (CCCC) ^3^
B-C_4_H_6_	neutral	3.43	1.492	1.386	139	
	anion	3.69	1.552	1.354	143	
	cation	4.06	1.416	1.532	133	
B-(C_4_H_6_)_2_	neutral	4.49	1.586	1.334	145	54
	anion	5.58	1.599	1.357	136	90
	cation	8.18	1.563	1.311	139	0
B-C_4_H_8_	neutral	2.14	1.545	1.595	118	
	anion	2.64	1.640	1.513	116	
cis B-(C_4_H_8_)_2_	neutral	4.12	1.612	1.494	145 ± 3	62
	anion	5.95	1.589	1.553	136	90
	cation	6.61	1.623	1.462	153	0
trans B-(C_4_H_8_)_2_	neutral	4.07	1.553, 1.704	1.566, 1.446	136, 153	77
	anion	5.95	1.590	1.553	137	83
	cation	6.61	1.620	1.463	154	6
C_4_H_6_-B-C_4_H_8_	neutral	4.72	1.517, 1.695	1.372, 1.463	140; 151	94
	anion	5.70	1.588, 1.603	1.347, 1.565	139; 136	86
	cation	7.71	1.508, 1.690	1.345, 1.429	149; 158	4

^1^ middle CC unit (bound to B). ^2^ CCC bond angle in C_4_H_6_; CCCC axial-folding angle in C_4_H_8_. ^3^ twisting angle between two opposite middle C-C units.

## Data Availability

The data presented in this study are available on request from the corresponding author.
